# Gendered effects of pay for performance among family physicians for chronic disease care: an economic evaluation in a context of universal health coverage

**DOI:** 10.1186/s12960-019-0378-0

**Published:** 2019-05-31

**Authors:** Neeru Gupta, René Lavallée, James Ayles

**Affiliations:** 1 University of New Brunswick, 3 Bailey Drive, Fredericton, New Brunswick Canada; 2 New Brunswick Department of Health, 520 King Street, Fredericton, New Brunswick Canada

**Keywords:** Medical workforce, Family physicians, Pay for performance, Diabetes mellitus, Gender gap, Health economics

## Abstract

**Background:**

Despite increasing popularity among health organizations of pay for performance (P4P) for the provision of comprehensive care for chronic non-communicable diseases, evidence of its effectiveness in improving health system outcomes is weak. An important void in the evidence base is whether there are gendered differences in P4P uptake and in related outcomes amenable to healthcare improvement. This study assesses the gender-specific effects of P4P among family physicians on diabetes healthcare costs in a context of universal health coverage.

**Methods:**

We use population-based linked longitudinal administrative datasets on chronic disease cases, physician billings, hospital discharge abstracts, and physician and resident registries in the province of New Brunswick, Canada. We estimate the effects of introduction of a P4P scheme on excess public healthcare costs among cohorts of adult diabetes patients using propensity score-adjusted difference-in-differences regressions stratified by physician’s gender.

**Results:**

We observed greater male physician uptake of incentive payments, seemingly exacerbating gender gaps in professional remuneration. Regression results indicated P4P did not lead to improved outcomes in terms of preventing hospitalization costs among patients, only measurable increases in compensation for both the male and female physician workforce.

**Conclusions:**

While P4P was not attributed in this study to reduced hospital burden and enhanced sustainability of healthcare financing, incentive payments were found to be related to earning gaps by physician’s gender. Decision-makers should consider that benefits of P4P be monitored not only for patient metrics but also for provider metrics in terms of gender equality especially given feminization of primary care medical workforces.

## Background

Countries in all world regions and at all levels of development are striving to reach evidence-informed decisions on resource allocation while moving towards the Sustainable Development Goals of universal health coverage, reducing the burden of non-communicable and infectious diseases, and gender equality. Optimizing health system performance entails improving population health, enhancing patient care experiences, and reducing the per capita cost of care, but there is also increasing recognition that achieving the ultimate goal of an efficient, effective, and equitable health system requires improving the experience and work life of care providers [1]. Ironically, while health services are often considered inadequately responsive to women’s healthcare needs, they are also highly dependent on women as providers of care [2]. Women are increasingly predominant in the primary care medical workforce in many countries [3]. Yet, work is not a gender-equal opportunity for women and men [4]. Males, including those in medical and other high-paying occupations, have long earned more than their female counterparts [5, 6]. International recognition of gender bias in incentives and resources for health and social workers is increasingly leading to calls for evidence that extend understandings and best practices for change [7]. Little is known about whether existing human resources for health (HRH) resourcing levers are related to better workforce performance metrics from a gender equity perspective.

In particular, the prevalence of chronic non-communicable diseases (NCDs) such as diabetes mellitus is increasing rapidly around the world, a trend attributable in large part to population aging and to rising rates of overweight and obesity. The World Health Organization advocates the population and public health burden of many NCDs can be reduced through promotion of interventions for better prevention and control, including appropriate patterns of clinical practice and counseling in primary care [8]. To address the growing public health and clinical challenge, financial incentives for healthcare providers—also known as pay for performance or P4P—for the delivery of patient-centered care are increasingly common in many health organizations [9–11]. These schemes are highly diverse across countries and jurisdictions, with different financial rewards and implementation mechanisms. Evaluations of P4P on healthcare improvement have ranged from absent to highly beneficial effects, related in part to wide differences in design choices and context [12]. Some P4P schemes offer bonuses for routine compliance with guideline-informed NCD care (such as seen in Denmark and two Canadian provinces) [13–15], others for the achievement of clinical care targets (such as seen in Taiwan and the United Kingdom) [16, 17]. Some offer higher bonuses for providers working in rural and remote areas (such as seen in Australia) [18].

We are unaware of any P4P programs accounting for physicians’ gender and other individual characteristics (aside from practice location) that may limit opportunities, real or perceived, for professional incentives. We are further unaware of any comprehensive analyses of the effects of workforce feminization on P4P metrics. This study aims to address this knowledge poverty by presenting a gendered evaluation of a P4P scheme for diabetes care among family physicians in the province of New Brunswick, Canada. First, we ask: was the uptake of P4P different by the physician’s gender? Second, we ask: did the introduction of P4P result in lower healthcare costs among the adult population with diabetes according to the physician’s gender? We used linked administrative datasets to address the two research questions in this context of universal health coverage.

## Methods

### Study setting

One of Canada’s smaller provinces, New Brunswick, represents 2.1% of the national population. This context is characterized by a relatively large rural population (48% rural compared to a national average of 19%), rapid aging (median age of 45.7 years versus 41.2 years nationally), and lower socioeconomic status (17.1% prevalence of after-tax low income versus 14.2% nationally) [19]. Over one third (37.5%) of the adult population are obese, a proportion significantly higher than the national average (26.9%) [20]. Most New Brunswickers (93.6%) report having a regular healthcare provider [20]. As with other Canadian jurisdictions, medically necessary physician and hospital services are covered for all eligible residents by the provincial government’s healthcare program. The majority of physicians are paid on fee-for-service (FFS) basis.

Fueled by multiple demographic, lifestyle, and socio-environmental factors, one in 10 New Brunswickers (9.8%) have been diagnosed with type 1 or type 2 diabetes [21]. National chronic disease surveillance data indicate the age-standardized diabetes prevalence rate has remained significantly higher in New Brunswick than the national average during the past decade [22]. In 2011, the provincial government introduced a P4P scheme to enhance diabetes management in primary care. Financial incentives were offered to family physicians under FFS remuneration for the provision of a set of guideline-based diabetes care services throughout the year, including regular blood glucose and blood pressure tests, counseling for weight management (and smoking cessation as appropriate), and completion of or referral for other tests for detection and prevention of common complications (lipid profile, renal function test, foot exam, eye exam) [23]. Overall effectiveness of such investments was expected to be measured in terms of decreases in hospital stays by persons with diabetes [24].

### Data sources

We draw on linked longitudinal administrative datasets from the provincial health department covering chronic disease cases, physician billings, hospital discharge abstracts, and physician and resident registries. Thanks to single-payer universal health coverage, the datasets are considered population representative and virtually complete. Cases of diabetes (types 1 and 2) are identified through validated algorithms tracing individuals’ interactions with the healthcare system [25]. The physician billings dataset includes all medical claims for services rendered to New Brunswick residents, including payments among FFS physicians and shadow billings among alternative-funded physicians and nurse practitioners (that is, claims submitted for administrative purposes by practitioners who are paid a salary or who work under contract). The hospital discharge abstract database covers diagnoses and procedures for all in-patient stays. The physician registry contains information on practitioners’ primary remuneration type, while the resident registry captures data on patients’ insurance eligibility status and demographic characteristics.

### Statistical analysis

Following a descriptive analysis of P4P uptake by physicians’ gender, we apply a propensity score-adjusted difference-in-differences econometric model to estimate the impacts of P4P on healthcare costs, notably in terms of (i) physician costs, (ii) potentially avoidable hospitalization costs attributable to diabetes, (iii) potentially avoidable hospitalization costs for common comorbid conditions (e.g., hypertension, ischemic heart disease, chronic kidney disease), and (iv) total physician and hospital costs for cohorts of New Brunswickers with diabetes, by patient exposure to physician uptake of the P4P incentive. In this context of universal coverage, physician and hospital costs are an aggregate reflection of both the frequency and intensity of use of healthcare resources to meet essential medical needs.

The difference-in-differences regression model is detailed elsewhere, as part of an earlier “gender-blind” investigation of P4P effects [14]. In brief, the model evaluates the counterfactual of whether healthcare costs would have been lower for patients with diabetes if P4P had not been introduced. We track longitudinal data on our key outcomes from before and after the implementation of P4P, among both patients exposed to physician uptake of the incentive and those not exposed, with propensity score matching on an observed patient and provider characteristics at baseline [26–28]. Our study pools data spanning from the 2009–2010 to 2014–2015 fiscal years, a period of limited other transformative changes to primary care medical services for diabetes management, and of upward but roughly parallel trends before the introduction of P4P in physician costs by remuneration model [14]. We estimate the difference between the pre-P4P cost trend projected forward and the post-P4P actual averaged cost. In this analysis, we take the model further by stratifying all analyses by the physician’s gender.

To assess both the short- and medium-term effects of P4P, we distinguish two patient cohorts: (i) a baseline cohort of residents 35 and older ever diagnosed with diabetes (type 1 or type 2) before the study period and (ii) a cohort of residents 35 and older newly diagnosed with diabetes (assumed type 2 only) in the year prior to introduction of the P4P scheme. Patients’ aggregate healthcare costs are measured in logged 2009 constant Canadian dollars [14]. The regression analyses were conducted in the Stata statistical software with the “diff” package, with propensity scores generated at the first stage and weighted bootstrap estimation of coefficients and standard errors at the second stage [28]. A difference-in-differences estimator less than zero suggests the introduction of P4P was of substantive importance in lowering costs. We set the significance level at *p* < 0.01.

### Research approach

The objective of this research is to strengthen the evidence base on gender and P4P to inform equitable and sustainable health financing policy decisions. In accordance with identified best practices for success in strengthening capacities among evaluators, researchers, and funders in health research [29–31], this investigation secured local institutional leadership and ownership of the results from the onset. To facilitate effective translation of knowledge into action, partners at the provincial health department have been active contributors throughout the research project: study design and selection of key outcome metrics, management and analysis of data, interpretation of findings, and dissemination of results.

## Results

### Descriptives of the patient and provider populations

**Fig. 1:**
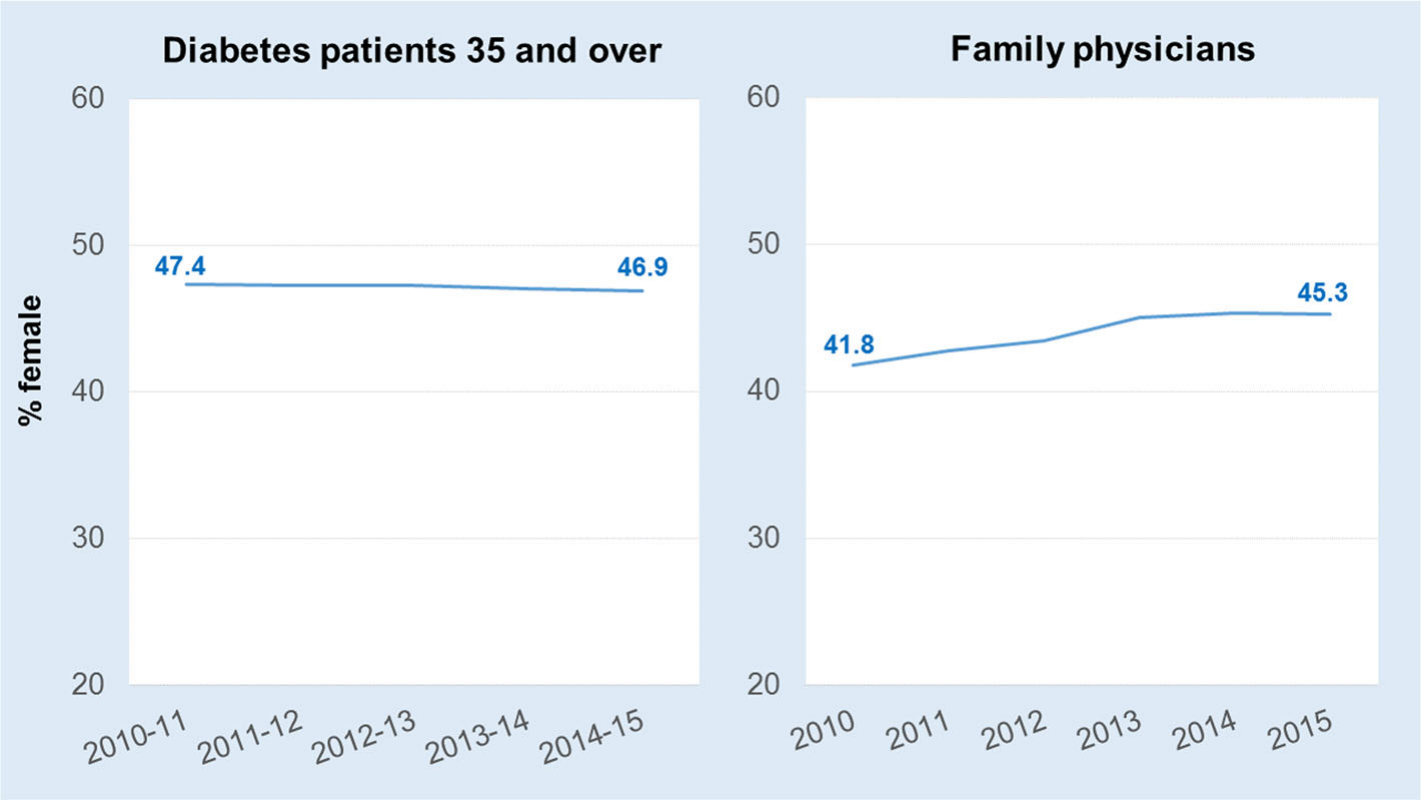
Sex distribution (%) of the diabetes patient and family physician populations, New Brunswick (Canada), 2010 to 2015

In New Brunswick, 13.6% of the adult population aged 35 years and over were living with diabetes in 2014–2015. Reflecting established epidemiological patterns, females were somewhat under-represented (47%) among patients with diabetes (Fig. 1). The proportion of the family physician workforce who were female increased to 45% in 2015 from 42% 5 years earlier, a pattern of feminization roughly echoing the national trend [32].

**Fig. 2:**
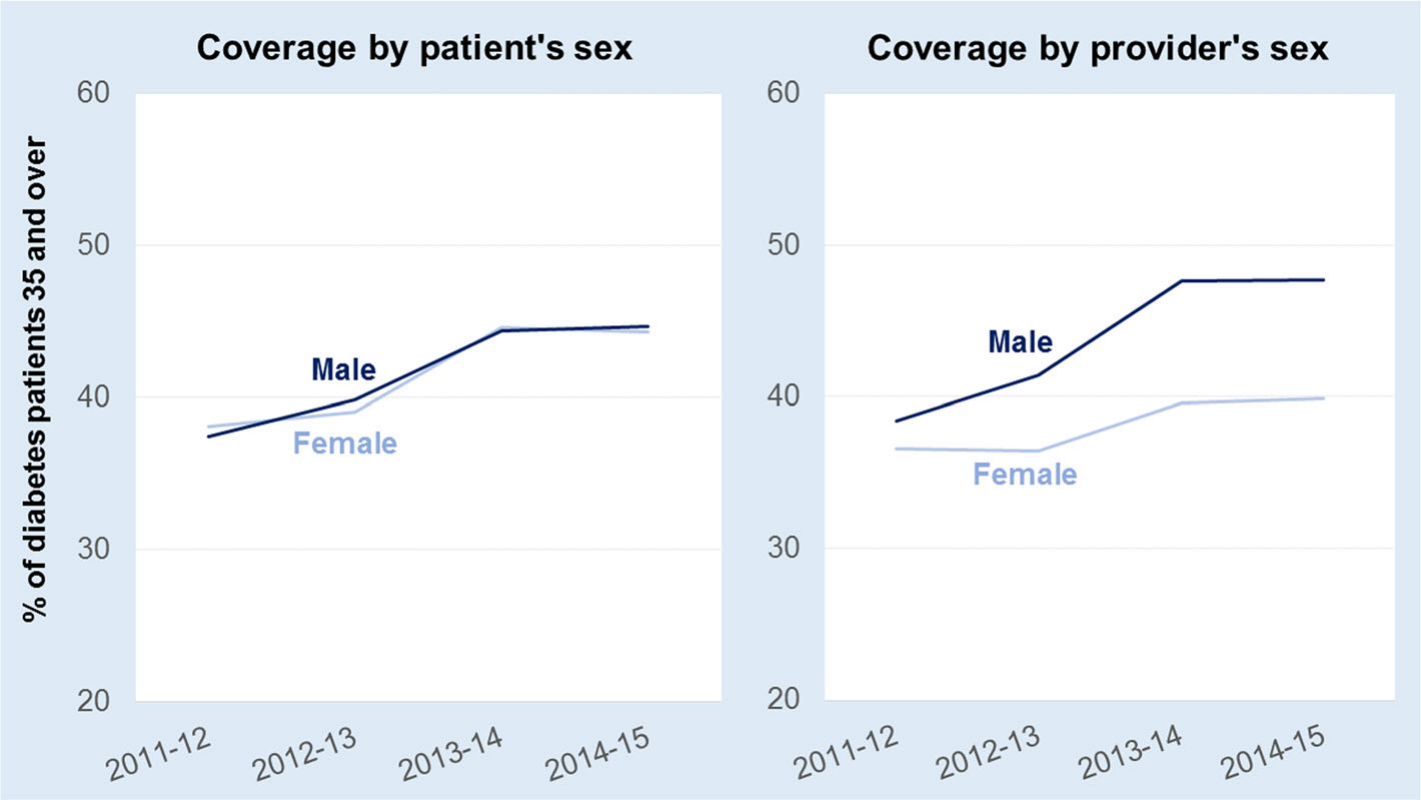
Coverage rate (%) of P4P for diabetes care by the patient’s sex and the sex of the patient’s provider, New Brunswick (Canada), 2011–2012 to 2014–2015

The coverage rate of P4P was less than half (44%) of adults 35 and older with diabetes in 2014–2015. There was no discernible difference in P4P coverage among male versus female patients (Fig. 2). However, over time patients of male providers were increasingly more likely to have received incentivized care (that is, their provider had claimed the financial incentive) compared to patients of a female provider.

Female providers have been under-represented in terms of billing claims for the incentive for diabetes care. Although females represent half (51%) of family physicians of diabetes patients, only 36% of P4P claims were submitted by a female provider (Table 1). This gender gap mirrors the tendency for female providers to submit less in FFS claims overall (valued 25% less) than their male counterparts. Moreover, P4P incentives account for a larger share (albeit remaining small in absolute terms) of total compensation among male versus female providers.

**Table 1:** Selected characteristics of family physicians of diabetes patients, by provider’s sex

	% of the family physician workforce	% of P4P claims	Mean no. of P4P claims (2011–2015)	Mean total FFS amount claimed (2015)	P4P amount as % of total FFS amount
Female providers	51	36	236	$190 120	.12
Male providers	49	64	427	$254 350	.17

Source: Linked provincial administrative health datasets

### Patient-level impacts of P4P by provider’s gender

As seen in Table 2, among the baseline cohort of adults living with diabetes, the effects of P4P on public healthcare costs did not generally differ by physician’s gender. While the numeric values of the coefficients are not intrinsically meaningful, the difference-in-differences estimators indicate that the trends in potentially avoidable hospitalization costs for diabetes (model 2) and for other common comorbidities (model 3) were not significantly different among the patient group exposed to physician P4P uptake compared to the non-incentive group—and this for patients of either female or male providers. In other words, there is no evidence 4 years after its introduction that P4P for diabetes care decreased the burden on the hospital system. On the other hand, significantly higher physician workforce costs (model 1) and all-cause healthcare costs (model 4) were attributed to the P4P scheme, for patients of both female and male providers.

**Table 2:** Difference-in-differences matching regression estimates (and associated standard errors) of the effects of P4P for diabetes care on healthcare costs, by provider’s sex

	(1)	(2)	(3)	(4)
	Physician workforce costs	Hospital costs for diabetes	Hospital costs for comorbid conditions	All-cause healthcare costs
Baseline cohort: adult patients diagnosed with diabetes before the study period				
Female providers	0.218* (0.024)	0.049 (0.021)	0.082 (0.035)	0.265* (0.031)
Male providers	0.152* (0.011)	0.002 (0.014)	− 0.011 (0.024)	0.188* (0.018)
Newly diagnosed type 2 diabetes adult patient cohort				
Female providers	0.207* (0.059)	− 0.059 (0.048)	− 0.200 (0.106)	0.184 (0.094)
Male providers	0.179* (0.042)	− 0.091 (0.041)	− 0.108 (0.089)	0.169 (0.074)

Note: *p < 0.01. Coefficients (and robust standard errors) calculated using propensity score difference-in-differences estimation. Outcomes are log healthcare costs in inflation-adjusted Canadian dollars. Matching variables include patient’s age group, sex, urban/rural residence, comorbid conditions (hypertension, ischemic heart disease), and physician practice variables (remuneration model, practice size). Baseline cohort includes adults aged 35 and over diagnosed with diabetes before the period of observation (*N* = 42 896). Newly diagnosed cohort includes adults 35 and over diagnosed with diabetes in the year before the introduction of the P4P scheme (*N* = 6656). Models are split by the sex of the patient’s most responsible provider

Source: Linked provincial administrative health datasets

Among the cohort of newly diagnosed type 2 diabetes patients, we again observe significantly higher physician workforce costs (model 1) among the incentive group attributable to higher post-P4P medical service claims among female and male providers. There is some indication of a trend towards lower preventable hospital costs for diabetes and its common comorbidities (models 2 and 3), but the results are not statistically significant. The full set of coefficients from the propensity-score adjusted difference-in-differences models can be found in the Appendix.

## Discussion

Physician services account for a significant proportion of health spending in most countries; in Canada, for example, physician services account for 15.1% of the total [33]. Financial incentives are increasingly being adopted across health agencies as a means to mitigate cost growth through better disease prevention and management, but there is little evidence on the implications for wage equity among healthcare providers. Our assessment through a gender lens of a pay for performance scheme among family physicians for diabetes care in a Canadian province, a context of high diabetes burden and universal health coverage, presents mixed results. Greater male physician uptake of incentive payments was found, seemingly exacerbating gender gaps in professional earnings among providers with fee-for-service remuneration. There is some evidence that female physicians tend to spend more time with each patient and deal with multiple health issues during a given visit compared to their male counterparts [3]. Such gendered differences in clinical practice patterns may drive earnings inequality under traditional FFS arrangements.

On the other hand, the present results using econometric evaluation methods indicate that the introduction of P4P in primary care has not yet led to preventing excess hospitalization costs among patients of either male or female providers. Rather, incentive payments led to measurable increases only in compensation for both the male and female physician workforces. Such findings are consistent with other Canadian studies, but which were not stratified by physician’s gender [14, 15]. They also reflect the wider deficiency in the availability of evidence to support the use of financial incentives to improve the quality of primary care [34].

Women may respond less to P4P for a range of social, cultural, and psychological reasons [6]. Research has also suggested that diabetes patients of female physicians are more likely to receive the guideline-based number of glycosylated hemoglobin tests compared to patients of male physicians, and this both before and after the implementation of P4P [35]. This raises the crucial question as to whether increasing numbers of women in medicine may drive change in patient-centered care without P4P. Research to date has been hampered by a lack of availability of linkable datasets that are sex-disaggregated, span over multiple years, and allow consideration of confounding factors such as practice type and numbers of patients seen, coupled with contextual information on pay policies and institutional systems [36]. This is, to our knowledge, the first study from a system of single-payer health insurance that directly seeks to examine gendered effects of P4P within the medical workforce. The context of single-payer universal coverage means we minimize the risk of unintended consequences of female health professionals potentially sorting out of health organizations with a strong performance pay component or having other characteristics that may be less attractive to women [6].

A key strength of our study was the use of population-based linked longitudinal datasets covering all cases of diabetes, physician service claims, and hospitalizations disaggregated by sex. Certain limitations should be noted, including exclusions to healthcare costing for emergency department visits, pharmaceuticals, and diabetes education and residential care by other non-medical health professionals. While we were able to control for patients’ sex, age, and certain comorbidities (hypertension, heart disease) in the statistical matching technique, the administrative data lacked information on obesity, tobacco use, and other modifiable risk factors amenable to primary care response. We were further lacking information on providers’ working hours, only claims for services rendered. The expanded use of electronic medical records, which remained relatively limited in New Brunswick in the period under consideration of this study, should help strengthen future research and policy monitoring with timely and comprehensive information.

## Conclusions

Heterogeneity of financial incentives for health professionals across jurisdictions and of related evaluation methods means the evidence base on physician responses to P4P remains weak. Previous systematic reviews have reported insufficient evidence of the effectiveness of P4P in improving different indicators of healthcare processes, costs, and outcomes [9, 12, 34]. Our evaluation from a context of universal health coverage indicated P4P uptake for chronic disease care differed by physician’s gender, coinciding with negligible beneficial impacts over the period of observation on patients’ risk of preventable hospitalization. We aimed to promote gender mainstreaming as an overlooked leadership tool to maximize the impact of financing options to support health system goals. An underlying objective was to mobilize evidence-informed discourse and inquiry to enhance understanding of whether gender-blind provider remuneration structures may unintentionally reinforce gender gaps. For example, many countries have sex-specific retirement ages, but we are unaware of any HRH financing models that consider the method of pay as regards gender earning differences within a given cadre. We propose that more research is needed using sex-disaggregated analyses from various settings to determine if there are true differences between male and female providers in the uptake of P4P and related outcomes amenable to healthcare improvement and promotion of gender equality in the health workforce.

## Appendix

**Table 3:** Coefficients (and associated standard errors) from the propensity-score adjusted difference-in-differences regressions for the effects of pay for performance on healthcare costs among adult patients diagnosed with diabetes before the study period (baseline cohort), by provider’s sex

	(1)		(2)		(3)		(4)	
	Physician workforce costs		Hospital costs for diabetes		Hospital costs for comorbid conditions		All-cause healthcare costs	
	Exposed group	Control group	Exposed group	Control group	Exposed group	Control group	Exposed group	Control group
1. Female providers								
Pre-P4P	6.266	6.319	0.099	0.192	0.366	0.629	6.485	6.741
Difference	− 0.053** (0.016)		− 0.092** (0.018)		− 2.63** (0.023)		− 0.256** (0.022)	
Post-P4P	6.503	6.339	0.100	0.144	0.411	0.591	6.763	6.754
Difference	0.165** (0.015)		− 0.044** (0.014)		− 0.181** (0.023)		0.009 (0.019)	
Diff-in-diff	0.218** (0.024)		0.049* (0.021)		0.082* (0.035)		0.265** (0.031)	
2. Male providers								
Pre-P4P	6.229	6.321	0.108	0.180	0.405	0.660	6.462	6.760
Difference	− 0.093** (0.010)		− 0.071** (0.010)		− 0.255** (0.018)		− 0.298** (0.015)	
Post-P4P	6.460	6.400	0.118	0.187	0.444	0.710	6.726	6.837
Difference	0.059** (0.008)		−0.069** (0.009)		− 0.266** (0.018)		− 0.111** (0.011)	
Diff-in-diff	0.152** (0.011)		0.002 (0.014)		−0.011 (0.024)		0.188** (0.018)	

Note: ***p* < 0.01, **p* < 0.05. Coefficients (with a bootstrap estimation of robust standard errors in parentheses) calculated using propensity score difference-in-differences estimation. Outcomes are log healthcare costs in inflation-adjusted Canadian dollars. Matching variables include patient’s age group, sex, urban/rural residence, comorbid conditions (hypertension, ischemic heart disease), and physician practice variables (remuneration model, practice size). Baseline cohort includes adults aged 35 and over diagnosed with diabetes before the period of observation (*N* = 42 896). Models are split by the sex of the patient’s most responsible provider

Source: Linked provincial administrative health datasets

**Table 4:** Coefficients (and associated standard errors) from the propensity-score adjusted difference-in-differences regressions for the effects of pay for performance on healthcare costs among adults diagnosed with diabetes in the year before the introduction of the P4P scheme (newly diagnosed cohort), by provider’s sex

	(1)		(2)		(3)		(4)	
	Physician workforce costs		Hospital costs for diabetes		Hospital costs for comorbid conditions		All-cause healthcare costs	
	Exposed group	Control group	Exposed group	Control group	Exposed group	Control group	Exposed group	Control group
1. Female providers								
Pre-P4P	6.166	6.171	0.145	0.106	0.464	0.423	6.385	6.488
Difference	− 0.005 (0.048)		0.039 (0.041)		0.041 (0.076)		− 0.103 (0.084)	
Post-P4P	6.249	6.046	0.030	0.050	0.223	0.383	6.426	6.345
Difference	0.203** (0.040)		− 0.020 (0.025)		− 0.159* (0.069)		0.081 (0.057)	
Diff-in-diff	0.207** (0.059)		− 0.059 (0.048)		− 0.200 (0.106)		0.184* (0.094)	
2. Male providers								
Pre-P4P	6.089	6.107	0.163	0.123	0.478	0.582	6.331	6.458
Difference	− 0.019 (0.042)		0.040 (0.036)		− 0.104 (0.074)		− 0.127* (0.063)	
Post-P4P	6.207	6.046	0.054	0.105	0.267	0.479	6.387	6.344
Difference	0.161** (0.026)		−0.051* (0.020)		− 0.212** (0.051)		0.042 (0.038)	
Diff-in-diff	0.179** (0.042)		− 0.091* (0.041)		− 0.108 (0.089)		0.169* (0.074)	

Note: ***p* < 0.01, **p* < 0.05. Coefficients (with a bootstrap estimation of robust standard errors in parentheses) calculated using propensity score difference-in-differences estimation. Outcomes are log healthcare costs in inflation-adjusted Canadian dollars. Matching variables include patient’s age group, sex, urban/rural residence, comorbid conditions (hypertension, ischemic heart disease), and physician practice variables (remuneration model, practice size). Newly diagnosed cohort includes adults 35 and over diagnosed with diabetes in the year before the introduction of the P4P scheme (*N* = 6 656). Models are split by the sex of the patient’s most responsible provider

Source: Linked provincial administrative health datasets

## Abbreviations

FFS: Fee-for-service
HRH: Human resources for health
NCD: Non-communicable disease
P4P: Pay for performance

## Authors’ information

NG is an associate professor at the University of New Brunswick (Fredericton, New Brunswick, Canada). RL and JA are the manager of Quantitative Services and director of Health Analytics, respectively, at the New Brunswick Department of Health (Fredericton, New Brunswick, Canada).

## References

[1] Bodenheimer T, Sinsky C (2014). From triple to quadruple aim: care of the patient requires care of the provider. Ann Fam Med.

[2] Lavallée R, Hanvoravongchai P, Gupta N, Dal Poz MR, Gupta N (2009). Use of population census data for gender analysis of the health workforce. Handbook on monitoring and evaluation of human resources for health.

[3] Hedden L, Barer ML, Cardiff K (2014). The implications of the feminization of the primary care physician workforce on service supply: a systematic review. Hum Resour Health.

[4] Tannenbaum C, Voss P (2016). Gender, work, and aging. Can J Aging.

[5] Baker LC (1996). Differences in earnings between male and female physicians. N Engl J Med.

[6] Bandiera O, Fischer G, Prat A, Ytsma E (2017). Do women respond less to performance pay? Building evidence from multiple experiments. CEPR discussion paper no. DP11724.

[7] Dhatt R, Keeling A, Schiegg N, Thompson K. A call to action on gender equality in global health. Devex [online content, 26 Jan 2018]. https://www.devex.com/news/opinion-a-call-to-action-on-gender-equality-in-global-health-91957. Accessed 21 May 2019.

[8] World Health Organization (2014). Global status report on noncommunicable diseases.

[9] de Bruin SR, Baan CA, Struijs JN (2011). Pay-for-performance in disease management: a systematic review of the literature. BMC Health Serv Res.

[10] Elovainio R (2010). Performance incentives for health in high-income countries: key issues and lessons learned. World Health Report (2010) Background Paper No. 32.

[11] Scheffler RM (2010). Pay for performance (P4P) programs in health services: what is the evidence? World Health Report (2010) Background Paper No. 31.

[12] Van Herck P, De Smedt D, Annemans L (2010). Systematic review: effects, design choices, and context of pay-for-performance in health care. BMC Health Serv Res.

[13] Rudkjøbinga A, Vrangbaek K, Birk HO, Andersen JS, Krasnik A (2015). Evaluation of a policy to strengthen case management and quality of diabetes care in general practice in Denmark. Health Policy.

[14] Gupta N, Lavallée R, Ayles J. Effects of pay-for-performance for primary care physicians on preventable diabetes-related hospitalization costs among adults in New Brunswick: a quasi-experimental evaluation. Can J Diabetes. 2018; [in press]. 10.1016/j.jcjd.2018.11.006. 10.1016/j.jcjd.2018.11.006 30679059

[15] Lavergne MR, Law MR, Peterson S (2016). A population based analysis of incentive payments to primary care physicians for the care of patients with complex disease. CMAJ.

[16] Hsieh HM, Gu SM, Shin SJ (2015). Cost-effectiveness of a diabetes pay-for-performance program in diabetes patients with multiple chronic conditions. PLoS One.

[17] Pandya A, Doran T, Zhu J (2018). Modelling the cost-effectiveness of pay-for-performance in primary care in the UK. BMC Med.

[18] Greene J (2013). An examination of pay-for-performance in general practice in Australia. Health Serv Res.

[19] Statistics Canada. Table: New Brunswick (province) and Canada (country). Census Profile, 2016 Census [online database]. Statistics Canada Catalogue No. 98–316-X2016001. Ottawa. Released 29 Nov 2017. https://www12.statcan.gc.ca/census-recensement/2016/dp-pd/prof/index.cfm. Accessed 20 Nov 2018.

[20] Statistics Canada. Table 13-10-0096-01: Canadian health characteristics, annual estimates [online database]. Ottawa. https://www150.statcan.gc.ca/t1/tbl1/en/cv.action?pid=1310009601. Accessed 20 Nov 2018.

[21] Gupta N (2017). Charting the progression of diabetes mellitus in New Brunswick: rates, correlates, and implications for accountability in public policy. J New Brunswick Stud.

[22] Department of Health (2016). Profiles on health: Diabetes mellitus in New Brunswick.

[23] Department of Health (2016). New Brunswick physicians’ manual.

[24] Department of Health (2011). A comprehensive diabetes strategy for New Brunswickers, 2011–15.

[25] Chen G, Khan N, Walker R, Quan H (2010). Validating ICD coding algorithms for diabetes mellitus from administrative data. Diabetes Res Clin Pract.

[26] Heckman J, Ichimura H, Todd P (1998). Matching as an econometric evaluation estimator. Rev Econ Stud.

[27] Stuart EA, Haiden A (2014). Using propensity scores in difference-in-differences models to estimate the effects of a policy change. Health Serv Outcomes Res Methodol.

[28] Villa JM (2016). Simplifying the estimation of difference-in-differences treatment effects. Stata J.

[29] ESSENCE on Health Research (2016). Six practices to strengthen evaluation of global health research for development.

[30] Gagliardi AR, Whitney B (2016). Integrated knowledge translation (IKT) in health care: a scoping review. Implement Sci.

[31] Cargo M, Mercer SL (2008). The value and challenges of participatory research: strengthening its practice. Annu Rev Public Health.

[32] Canadian Institute for Health Information (2018). Supply, distribution and migration of physicians in Canada, 2016: historical data.

[33] Canadian Institute for Health Information (2018). National health expenditure trends, 1975 to 2018.

[34] Scott A, Sivey P (2011). The effect of financial incentives on the quality of health care provided by primary care physicians. Cochrane Database Syst Rev.

[35] LeBlanc E, Bélanger M (2016). Influence of a pay-for-performance program on glycemic control in patients living with diabetes by family physicians in a Canadian province. Can J Diabetes.

[36] Global Health Workforce Network (2018). Gender and equity in the health and social care workforce: consultative draft report. Gender equity hub working paper.

